# Breath-held 3D coronary vessel wall imaging with dual-density spiral acquisition and parallel imaging

**DOI:** 10.1186/1532-429X-14-S1-W62

**Published:** 2012-02-01

**Authors:** Meihan Wang, Michael Salerno, Christopher M Kramer, Craig H Meyer

**Affiliations:** 1 Biomedical Engineering, University of Virginia, Charlottesville, VA, USA; 2 Medicine, University of Virginia, Charlottesville, VA, USA; 3 Radiology, University of Virginia, Charlottesville, VA, USA

## Summary

We have developed a single breath-hold 3D sequence with dual density spiral readout and parallel imaging for coronary vessel wall studies.

## Background

MR coronary wall imaging requires high spatial resolution to detect atherosclerotic plaque. A number of methods have been developed to acquire the necessary resolution with sufficient SNR [1, 2]. However, the scan time of those sequences is usually too long for one breath hold. In that case, navigator and low resolution images are obtained to correct for patient motion. The resultant images can suffer from image blur if there is inadequate motion correction. In this study, we will present an efficient single breath-hold segmented 3D gradient-echo sequence using dual-density spiral readouts and parallel imaging to overcome these limitations. Double inversion recovery is also used to suppress blood signal and produce high contrast coronary images for coronary artery wall.

## Methods

During each heartbeat, after double inversion recovery preparation and fat saturation pulses, a 3D GRE sequence is executed. The sequence is modified to use 8ms dual-density spiral gradient to obtain high resolution images. Centric ordering is used in the kz direction. The sequence was tested on the right coronary artery of healthy volunteers using a Siemens 3T scanner with a 32-channel coil. The imaging protocol was as follows: FOV = 350mm, flip angle = 30°, in-plane resolution = 0.9×0.9mm, slice thickness = 5mm, no. of slices = 8, TE = 2ms, TR = 1R-R interval, no. of interleaves = 72. The in-plane interleaves are undersampled by a factor of 4. The total acquisition time is then approximately 18 heartbeats, which is short enough for one breath-hold.

The images are reconstructed using SPIRiT[3], which is an autocalibrated parallel imaging technique. With dual-density readout, the center k-space is fully sampled for calibrating the convolution kernel for final reconstruction. The unaliasing step is performed iteratively until aliasing is removed.

## Results

**Figure 1:**
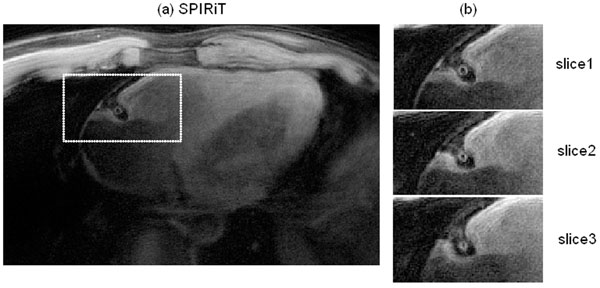
Right coronary artery wall images reconstructed by SPIRiT. Three images from the 3D dataset are shown on the right.

In the SPIRiT image (Fig1a), most of the aliasing is removed, although the tradeoff the enhancement of noise in some area. Coronary wall area is zoomed in for better display in Fig1b. Blood signal is sufficiently suppressed over the 3D volume.

## Conclusions

We have combined dual density spiral and parallel imaging in 3D GRE sequence to acquire high resolution coronary vessel wall images. The acquisition is completed in one breath hold to avoid motion artifact. SPIRiT is used to remove the aliasing and reconstruct final images.

## Funding

This study was funded by NIH R01 HL079110 and Siemens Medical Solutions.

